# EGFR外显子20插入突变：研究现状与治疗新策略

**DOI:** 10.3779/j.issn.1009-3419.2024.106.19

**Published:** 2024-08-20

**Authors:** Mengwei TIAN, Na WANG, Zhanjun DOU, Xia SONG, Xia ZHANG

**Affiliations:** ^1^030001 太原，山西医科大学第二临床医学院（田梦薇，王娜）; ^1^The Second Clinical Medical College of Shanxi Medical University, Taiyuan 030001, China; ^2^030013 太原，山西省肿瘤医院/中国医学科学院肿瘤医院山西医院/山西医科大学附属肿瘤医院呼吸科（窦占军，宋霞，张霞）; ^2^Department of Respiratory, Shanxi Province Cancer Hospital/Shanxi Hospital Affiliated to Cancer Hospital, Chinese Academy of Medical Sciences/Cancer Hospital Affiliated to Shanxi Medical University, Taiyuan 030013, China

**Keywords:** 肺肿瘤, 表皮生长因子受体, 外显子20插入突变, 靶向治疗, Lung neoplasms, Epidermal growth factor receptor, Exon 20 insertion mutation, Targeted therapy

## Abstract

作为非小细胞肺癌（non-small cell lung cancer, NSCLC）中重要的致癌驱动基因，表皮生长因子受体外显子20插入突变（epidermal growth factor receptor exon 20 insertion, EGFR ex20ins）具有独特蛋白构象，并且对传统EGFR酪氨酸激酶抑制剂（EGFR-tyrosine kinase inhibitors, EGFR-TKIs）原发耐药。近年来，靶向EGFR ex20ins的药物探索从未停止。莫博赛替尼与埃万妥单抗率先被美国食品药品监督管理局（Food and Drug Administration, FDA）获批用于EGFR ex20ins突变NSCLC患者，随后舒沃替尼等药物取得突破，联合治疗方案的探索也有所收获。多管齐下有望克服EGFR ex20ins耐药。因此，深入了解EGFR ex20ins的分子机制并评估新型药物的有效性与差异性至关重要。本文将对相关最新研究成果进行全面总结，以期为EGFR ex20ins突变患者精准治疗提供有价值的参考。

## Abstract (en)

In non-small cell lung cancer (NSCLC), as an improtant oncogenic driver gene, epidermal growth factor receptor exon 20 insertion (EGFR ex20ins) has a unique protein structure and is primarily drug-resistant to traditional EGFR-tyrosine kinase inhibitors (EGFR-TKIs). In recent years, exploration of targeted therapy for EGFR ex20ins has never stopped. Firstly Mobocertinib and Amivantamab obtained approval from U.S. Food and Drug Administration (FDA) for EGFR ex20ins mutant NSCLC patients, then other drugs, such as Sunvozertinib, made breakthroughs and combination therapies also obtained gains. Multi-pronged measures are hopeful to overcome EGFR ex20ins drug resistance. As mentioned above, it’s definitely important to gain deeper understanding of molecular mechanism of EGFR ex20ins and assess effect and difference between novel drugs. This review is devoted to make a summary about newest achievement so to provide valuable reference about precise therapy for patients with EGFR ex20ins.

目前肺癌是我国致死率最高的癌症^[1]^，其主要病理类型为非小细胞肺癌（non-small cell lung cancer, NSCLC），约占85%^[2]^。表皮生长因子受体（epidermal growth factor receptor, EGFR）是NSCLC最常见的致癌驱动基因之一^[3]^。EGFR外显子19框内缺失（EGFR exon 19 inframe deletion, EGFR ex19del）和EGFR外显子21 L858R改变（EGFR exon 21 L858R alterations, EGFR ex21L858R）是位居前两位的突变亚型，约占90%^[4]^，又称EGFR经典突变。靶向治疗已被证明是EGFR经典突变的最佳疗法^[5]^。然而，EGFR第三大突变亚型即EGFR外显子20插入突变（EGFR exon 20 insertion, EGFR ex20ins）具有与EGFR经典突变不同的独特构象变化，并且对传统EGFR-酪氨酸激酶抑制剂（EGFR-tyrosine kinase inhibitors, EGFR-TKIs）原发耐药^[6]^。与EGFR经典突变相比，EGFR ex20ins突变患者的预后更差^[7]^。近年来，除了对已批准的EGFR-TKIs进行探索外，新兴的药物对EGFR ex20ins突变患者也展现了令人惊喜的疗效，靶向治疗取得重大突破。本文将重点对EGFR ex20ins的分子亚型、分子机制和新型药物的最新研究成果进行全面总结。

## 1 EGFR ex20ins的流行病学概述

EGFR ex20ins多见于无吸烟史、腺癌、女性以及亚裔患者^[8]^，相较EGFR经典突变，其年轻化趋势明显^[9]^。在中国NSCLC患者中，其突变频率为1.6%，占EGFR突变的3.5%^[10]^。在转移方面，最常见转移部位依次为骨转移、脑转移和肝脏转移^[9]^，研究^[11]^表明约39%的患者诊断时即有脑转移。除此之外，携带该突变患者的预后通常较差，中位无进展生存期（progression-free survival, PFS）仅为EGFR经典突变患者的一半^[9]^。

## 2 EGFR ex20ins的定义与分子作用机制

EGFR ex20ins发生于EGFR外显子20上，涉及3-21个碱基对的框内插入或复制，可被表征为EGFR酪氨酸激酶结构域中第762至774位氨基酸（amino acid, AA）（参与组成结构域中的αC螺旋和C螺旋后的环）间1-7个AA的插入或复制（图1），导致EGFR下游信号通路的异常激活和细胞生长、增殖的失调^[12]^。EGFR ex20ins插入位点和插入AA的不同造就了繁多的突变亚型和不同亚型间的异质性，其亚型分类可概括为αC螺旋内插入（AA762-766）、近环插入（AA766-772）和远环插入（AA772-774）三大类，在我国检测到了85种不同突变亚型，其中近环插入和远环插入突变的占比高达94.7%，最常见的三种分子亚型分别为A767_V769dup、S768_D770dup和N771_H773dup，分别占所有突变亚型的25.1%、17.6%和4.8%，以1-3个AA插入最常见^[13]^。此外，EGFR ex20ins常与EGFR扩增和TP53突变共存^[14]^，研究^[15,16]^发现存在TP53或EGFR扩增共突变的EGFR ex20ins患者与化疗和靶向治疗较短的PFS相关。

**图1:**
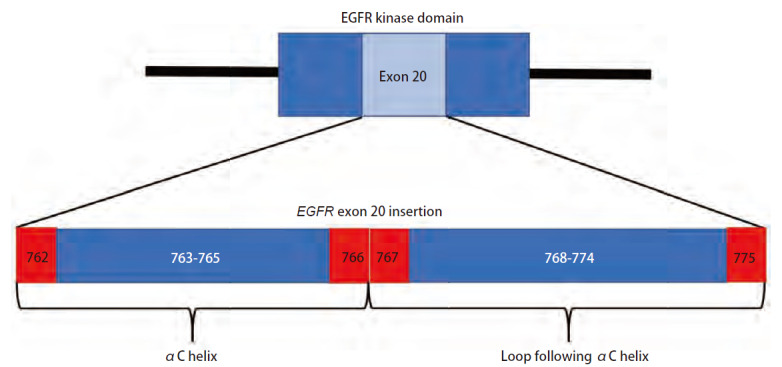
EGFR ex20ins的分子定位

在正常生理状态下，EGFR的激活表现为配体依赖型，配体与EGFR结合后EGFR完成二聚化，调动酪氨酸激酶结构域中的关键调节元件--αC螺旋向外旋转呈现出“αC-in”活性构象，打开腺嘌呤核苷三磷酸（adenosine triphosphate, ATP）结合口袋促进ATP结合位点自磷酸化^[6]^，激活下游信号通路促进细胞的生存、生长和增殖^[17]^。C螺旋的N末端与C末端和不同的环相连，EGFR ex20ins则多发生在C螺旋后的环上，导致C末端环的延长，将C螺旋“推”到活性构象，并且该过程并不依赖配体^[18]^。这种发生在C螺旋后环上的插入突变造成在ATP结合口袋形成“楔形结构”导致空间位阻，增加了EGFR-TKIs进入结合口袋内与靶点—ATP结合位点结合的难度^[19]^（图2）。相较于野生型EGFR（wild type EGFR, WT EGFR），EGFR ex20ins对ATP亲和力没有明显受损的同时对EGFR-TKIs的亲和力也并未明显升高，导致EGFR-TKIs与ATP竞争性结合ATP结合位点难度增加，否决了选择性靶向EGFR突变体优于WT EGFR的优势^[6,12,18,20,21]^。尽管提高药物浓度理论上可能提升疗效，但过高的血药浓度会波及WT EGFR导致脱靶毒性的产生，引发皮疹、腹泻等皮肤、黏膜和胃肠道的不良反应^[22]^。值得注意的是，部分特殊EGFR ex20ins亚型，如螺旋内插入突变EGFR A763_Y764insFQEA（诱导C螺旋向N端移位同时维持第762位AA活性位点结合、定向ATP的能力^[12]^）、770号氨基酸被甘氨酸取代的近环插入突变（增加激酶结构域单体亚基之间的吸引静电能保持EGFR的激活^[23]^）并不会导致空间位阻效应，所以传统EGFR-TKIs仍然可以对这些亚型发挥作用^[6,24,25]^。

**图2:**
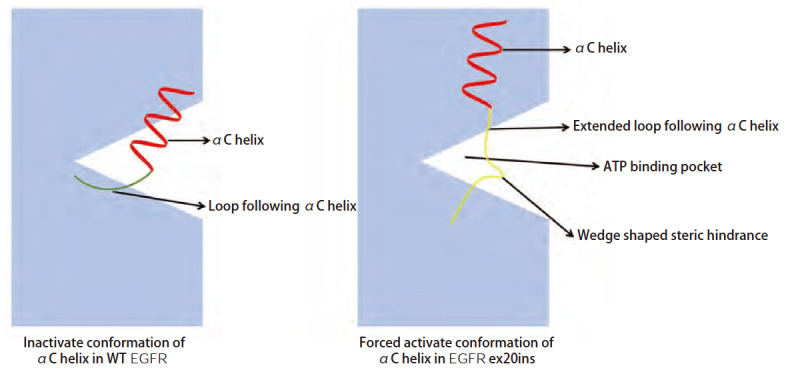
EGFR ex20ins的分子作用机制

在检测方法上，美国国立综合癌症网络（National Comprehensive Cancer Network, NCCN）指南^[26]^推荐下一代测序（next generation sequencing, NGS）而非实时聚合酶链式反应（real-time polymerasa chain reaction, RT-PCR）作为首选检测方法，概因RT-PCR相较NGS漏检率可达50%^[27]^。

## 3 EGFR ex20ins治疗新策略

目前临床常用的传统EGFR-TKIs、含铂方案化疗和免疫治疗对EGFR ex20ins NSCLC的整体疗效有限，其中含铂方案化疗表现相对较好，客观缓解率（objective response rate, ORR）为25.7%，PFS为5.6个月^[28]^。随着众多靶向药物的临床试验数据陆续披露，2024版中国临床肿瘤学会（Chinese Society of Clinical Oncology, CSCO）指南^[29]^的一线推荐治疗方案除含铂类化疗（IA类证据）外，以III级推荐新增埃万妥单抗联合含铂双药化疗作为EGFR ex20ins首个一线靶向疗法，后线治疗去除莫博赛替尼推荐意见，新增舒沃替尼作为含铂化疗后进展的EGFR ex20ins治疗方案，同埃万妥单抗以3类证据分别成为后线I和III级推荐治疗方案。EGFR ex20ins的两处治疗靶点可分为细胞内的EGFR-TKIs靶点与细胞外的单克隆抗体（monoclonal antibodies, mAbs）靶点（图3），本部分将对各靶向药物及其联合疗法进行详细叙述。

**图3:**
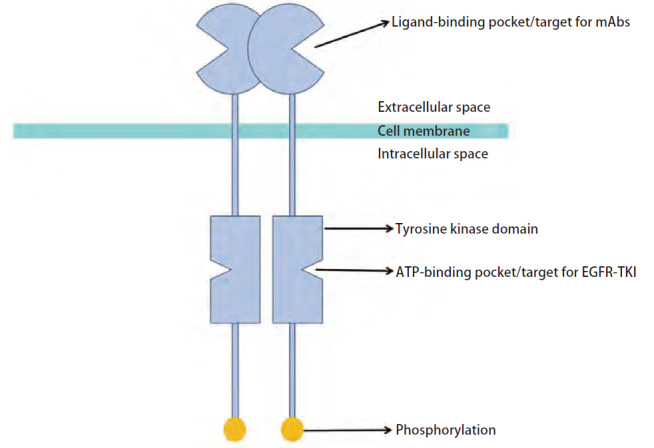
EGFR ex20ins靶向治疗的不同靶点

### 3.1 EGFR-TKIs单药疗法

经过几十年的发展，靶向EGFR胞内结构的EGFR-TKIs得到了广泛应用。一代EGFR-TKIs为ATP竞争性可逆EGFR-TKIs，依赖于EGFR突变体的低ATP亲和力，与ATP竞争性结合EGFR激酶结构域中的ATP结合位点^[19,30]^；二代EGFR-TKIs旨在与EGFR第797位半胱氨酸残基（cysteine 797, Cys797）（图4）结合形成共价不可逆复合物、抑制EGFR形成二聚体，克服EGFR突变体ATP亲和力升高导致的一代EGFR-TKIs耐药的问题，但半数抑制浓度（half maximal inhibitory concentration, IC_50_）下易波及WT EGFR产生脱靶毒性，治疗窗狭窄^[31,32]^；与二代EGFR-TKIs相比，三代EGFR-TKIs通过嘧啶骨架和多变的取代基，除了与Cys797形成共价结合外，相较二代EGFR-TKIs提高了对WT EGFR的选择性^[33]^。一、二代EGFR-TKIs仅在部分突变亚型上发挥了较好的抗肿瘤效应^[6,12,24,34]^，而三代EGFR-TKIs适用EGFR ex20ins突变更广。此外，真实世界研究（real-world study, RWS）^[10]^发现，相较于一线化疗，近环插入亚型接受二、三代EGFR-TKIs治疗获益更大，远环插入亚型一线化疗则表现更佳，提示EGFR ex20ins的治疗方案仍存在进一步细化的巨大空间和潜力。除外上述提及的已在临床中广泛应用的EGFR-TKIs，新研发的EGFR-TKIs药物通过特殊骨架或创新性取代基更加灵活的克服EGFR ex20ins靶点的空间位阻、增强靶点结合能力、避免抑制WT EGFR导致的脱靶毒性，也取得了不错的成果。

**图4:**
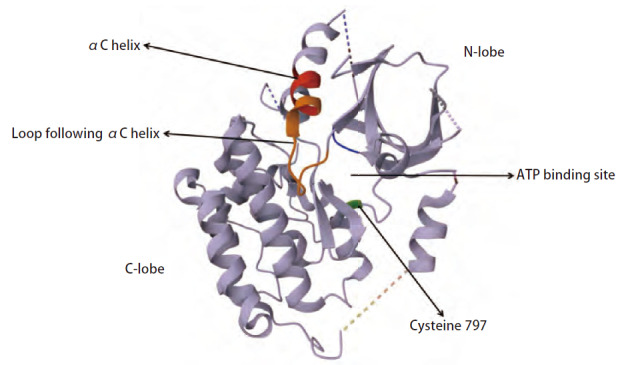
EGFR酪氨酸激酶结构域与EGFR-TKIs的结合位点（来源于蛋白质结构数据库PDB ID 1XKK）

#### 3.1.1 一代与二代EGFR-TKIs

多数EGFR ex20ins患者在接受一、二代EGFR-TKIs治疗后PFS仅有1.9-2.7个月^[35,36]^，因此临床实践中更倾向采用含铂类化疗^[28]^，但部分情况下一、二代EGFR-TKIs也可取得不亚于含铂类化疗的效果。RWS^[10]^表明，近环突变亚型可在二代EGFR-TKIs上获得优于含铂类化疗的PFS获益；部分特殊EGFR ex20ins亚型甚至在体外模型和临床研究中表现出对一、二代EGFR-TKIs的高敏感性和高临床获益。EGFR A763_Y764insFQEA在体外模型中对吉非替尼、阿法替尼等已批准的一、二代EGFR-TKIs均敏感^[12,34,37]^，阿法替尼的IC_50_甚至优于三代EGFR-TKIs^[38]^，靶向治疗疗效良好（ORR为62.5%，中位PFS为5.5个月）^[37]^。EGFR D770delinsGY则对二代EGFR-TKIs中的达可替尼和阿法替尼敏感（PFS为11-12.4个月）^[24]^，研究推测具有770号AA被甘氨酸取代特征的EGFR ex20ins亚型可能都对此类药物有敏感性^[6]^，但尚缺乏临床研究和大样本RWS支持该论点。

#### 3.1.2 三代EGFR-TKIs

##### 3.1.2.1 奥希替尼

奥希替尼通过结构优化有效抑制EGFR的磷酸化和下游信号通路，对EGFR ex20ins突变体表现出良好抗肿瘤活性^[39,40]^及较宽的治疗窗^[20]^。在小样本的回顾性研究^[41]^和LU17-19临床试验^[42]^中，标准剂量奥希替尼（80 mg, qd）疗效大相径庭（ORR：67.7% vs 0%；PFS：6.2 vs 3.5个月），不除外小样本导致了偏倚。而在探索高剂量奥希替尼（160 mg, qd）疗效的前瞻性研究POSITION 20^[43]^和ECOG-ACRIN 5162^[44]^中，一线和后线治疗疗效尚可（ORR: 24%-28%；PFS: 6.8-9.7个月），但治疗相关不良事件（treatment related adverse event, TRAE）高发，最常见的TRAE为腹泻（72%-76%）、疲劳（44%-67%）和血小板计数减少（20%-67%），20%-35%的患者经历了≥3级TRAE。一项中国大样本回顾性研究^[45]^则发现经奥希替尼治疗的EGFR ex20ins患者生存获益低（ORR：6.5%；PFS：2.3个月），且药物剂量对PFS无显著影响。故而在实际临床应用中，奥希替尼单药的疗效仍存在较大的不确定性。

##### 3.1.2.2 伏美替尼

伏美替尼通过引入独特的三氟乙氧基嘧啶结构获得宽广的安全窗口，药物本体和其活性代谢产物均表现出剂量依赖的抑瘤活性和高血脑屏障渗透性，易与血浆蛋白共价结合并在肺部富集延长起效时间^[46⇓-48]^。虽然其标准剂量为80 mg/d，但采用更高剂量或仍可同时保证安全性和疗效增益^[49]^。在Ib期FAVOUR临床试验^[50]^中，最高剂量（240 mg, qd）伏美替尼作为一线和后线疗法均有显著疗效（ORR：69.0% vs 50.0%；中位PFS：10.7 vs 7.0 个月）；后线治疗中降低剂量（160 mg, qd）疗效也未见明显下降（ORR：40.9%；中位PFS：5.8个月），同时在患者中观察到颅内活性。安全性上，高剂量下TRAE导致治疗中断率最高仅为4.2%。临床实践中，部分个案报道^[51,52]^表明了伏美替尼的可靠性，但也有RWS^[53]^表明二线治疗中化疗联合贝伐珠单抗方案获益优于伏美替尼，但并未注明伏美替尼剂量，另一项RWS^[54]^则给出了最高剂量（240 mg, qd）的ORR仅为37.7%。目前，一线对比伏美替尼与含铂方案化疗疗效的III期临床试验FURVENT正在进行中^[55]^。

##### 3.1.2.3 阿美替尼

阿美替尼创新性地将环丙基结构引入吲哚氮与Cys797靶点匹配，稳定发挥抑制EGFR磷酸化、下游信号转导、细胞增殖和诱导凋亡作用，在20和40 mg/kg浓度下携带EGFR ex20ins小鼠的肿瘤生长抑制率呈现剂量依赖（41% vs 64%）^[38]^。目前阿美替尼治疗罕见EGFR突变晚期NSCLC患者中的多中心III期临床试验 （NCT04951648）正在进行中，目前尚无EGFR ex20ins患者数据披露^[56]^。

#### 3.1.3 新研发的EGFR-TKIs

##### 3.1.3.1 莫博赛替尼

莫博赛替尼通过创新性异丙酯结构增强与EGFR ex20ins突变体的结合，抑制EGFR磷酸化以及下游信号传递^[22]^。在I/II期临床研究^[57,58]^中，莫博赛替尼在既往接受含铂类化疗EGFR ex20ins患者中ORR为28%，中位PFS为7.3个月，疗效不受突变亚型影响，无颅内活性数据披露。在安全性上，47%的患者出现≥3级TRAE，以腹泻（21%）、恶心（4%）、口腔炎（4%）最常见，但均可通过支持性治疗、剂量调整或停药进行管理^[58]^。莫博赛替尼因此获得国家药品监督管理局附条件批准^[59]^和美国食品药品监督管理局（Food and Drug Administration, FDA）加速批准^[60]^用于治疗铂类化疗失败的EGFR ex20ins晚期NSCLC。然而，对比一线莫博赛替尼与含铂化疗疗效的III期临床试验EXLAIM-2^[61]^中，莫博赛替尼较含铂化疗未见明显获益（ORR：32% vs 30%；PFS：9.59 vs 9.63个月），未能达到PFS主要研究终点，出现≥3级TRAE患者却较化疗有所增加（62% vs 53%），不符合加速批准/附条件批准的要求，最终莫博赛替尼在美国^[62]^与中国^[63]^主动退市，CSCO指南也将其I级推荐移除。

##### 3.1.3.2 舒沃替尼

舒沃替尼通过2-氨基嘧啶母环和苯胺基取代实现对EGFR ex20ins的高度亲和力和选择性^[64]^。I/II期临床试验^[64⇓-66]^中，舒沃替尼在经治EGFR ex20ins NSCLC患者中表现出宽广的治疗窗，100-300 mg/d剂量梯度间疗效良好且呈现递增趋势（ORR: 41.9%-61%），在既往接受含铂类化疗的患者中表现最佳（300 mg/d时ORR为61%），在基线脑转移、不同治疗线数和既往治疗方案情况下均能缩小靶病灶，进一步证明了舒沃替尼的出色疗效和广泛的应用潜力。在初治患者中，舒沃替尼（300 mg, qd）ORR高达77.8%^[67]^。安全性上，≥3级TRAE包括血肌酸磷酸激酶升高（17%）、腹泻（8%）和贫血（6%）。2023年舒沃替尼成功获批用于化疗失败后的EGFR ex20ins NSCLC患者^[68]^。目前，一项旨在比较一线舒沃替尼与标准铂类双药化疗在EGFR ex20ins NSCLC患者中疗效的III期临床试验（NCT05668988）正在进行中，数据尚未披露^[69]^。

##### 3.1.3.3 齐帕替尼

齐帕替尼具有可增强EGFR ex20ins选择性的新型吡啶嘧啶骨架，能有效抑制多种EGFR ex20ins突变亚型的细胞增殖，具有广谱抗EGFR活性和宽广的治疗窗^[70,71]^。在I/IIa期临床试验^[72]^中，使用最佳剂量齐帕替尼（100 mg, bid）时，经治EGFR ex20ins NSCLC患者ORR为41%，但疗效受突变亚型影响，近环突变较远环突变表现更好（ORR: 41.5% vs 22%）。齐帕替尼的血脑屏障穿透能力尚不明确^[72]^，但在1例基线脑转移患者中观察到颅内病灶缩小。安全性上，常见不良反应包括皮疹（80%）、甲沟炎（32%）和腹泻（30%），多为1-2级，可通过标准治疗管理。

##### 3.1.3.4 YK-029A

YK-029A通过占据EGFR中其他EGFR-TKIs无法到达的疏水口袋、与Cys797共价结合保持对EGFR ex20ins的选择性与稳定性，抑制EGFR磷酸化及抑制下游信号通路^[73]^。I期临床试验^[74]^招募了28例初治EGFR ex20ins NSCLC患者，接受YK-029A治疗后ORR可达73.1%，中位PFS为9.3个月，且在不同突变亚组中均有良好反应。安全性上，94.4%的患者出现TRAE，主要包括贫血（46.3%）、腹泻（38%）和皮疹（32.4%），27.8%的患者出现≥3级TRAE。目前，对比一线YK-029A与含铂化疗疗效的III期临床试验（NCT05767892）正在进行中，数据尚未披露^[73]^。

##### 3.1.3.5 PLB-1004

PLB-1004是一种具有单苯胺基嘧啶结构的新型EGFR-TKI^[75]^。在I期临床试验^[75]^中共纳入65例经治EGFR ex20ins NSCLC患者，分为11个剂量梯队（10 mg，qd至480 mg，qd），总体ORR可达57.7%，疾病控制率（disease control rate, DCR）为100%，但在最高剂量（480 mg, qd）队列中观察到由于毒性而导致的频繁剂量中断和剂量减少。安全性上，最常见的TRAE包括腹泻（75%）和皮疹（60%），分别有18%和11%为≥3级TRAE。目前评估PLB-1004用于既往接受过含铂化疗和/或免疫治疗EGFR ex20ins NSCLC患者的II期临床试验（NCT06015503）与一线对比含铂双药联合或不联合信迪利单抗的III期临床试验（NCT06281964）正在招募^[69]^。

##### 3.1.3.6 BEBT-109

BEBT-109是一种泛突变选择性EGFR-TKI，通过丙烯酰胺片段与Cys797不可逆结合，脱靶毒性低且能够穿过血脑屏障^[76]^。在纳入经治EGFR ex20ins NSCLC患者的Ia期临床试验^[77]^中展现了44.4%的ORR和8个月的中位PFS，近环突变和远环突变疗效相似（ORR: 40% vs 42.8%），1例EGFR A763_Y764FQEA阳性患者的脑部靶病灶出现缩小。安全性上，最常见的TRAE为腹泻（100%）、皮疹（66.7%）和贫血（61.1%），38.9%的患者出现≥3级TRAE，5.6%的患者因此停药。

### 3.2 mAbs单药疗法

mAbs具有与EGFR-TKIs不同的作用机制，其靶点位于细胞外，通过与天然配体竞争性结合EGFR胞外结构、形成抗体受体复合物并被内化降解导致肿瘤细胞表面EGFR的下调，同时触发抗体依赖和补体依赖的细胞毒效应激活先天免疫细胞发挥抗肿瘤效应。mAbs发挥抗肿瘤作用不受ATP影响，而EGFR-TKIs则通过阻断EGFR细胞内激酶结构域中的ATP结合位点发挥作用^[78⇓-80]^。mAbs独特作用机制使其显示出了广泛的应用前景。

#### 3.2.1 埃万妥单抗

埃万妥单抗是一种具有高亲和力、靶向EGFR与间质表皮转化因子（mesenchymal to epithelial transition factor, MET）的双特异性抗体^[79]^。在I期CHRYSALIS研究^[81]^中提出1400 mg（基线体重≥80 kg）与1050 mg（基线体重<80 kg）2种推荐剂量，在既往接受含铂类化疗的EGFR ex20ins NSCLC患者中显示出40%的ORR和8.3个月的中位PFS。主要TRAE为1-2级的皮疹（86%）、输液相关反应（66%）和甲沟炎（45%），4%的患者出现停药。2021年该药物已在美国获批用于治疗相关突变的患者^[82]^。

#### 3.2.2 西妥昔单抗/JMT101/耐昔妥珠单抗

JMT101、西妥昔单抗与耐昔妥珠单抗均为抗EGFR mAbs，三者并无单药靶向EGFR ex20ins的临床试验，仅在肿瘤生物模型中表现出靶向EGFR的抗肿瘤活性，其中JMT101在EGFR ex20ins异种移植模型中肿瘤生长抑制率可达60%^[80,83⇓-85]^。

### 3.3 联合治疗方案探索

正如上文所述，目前靶向EGFR ex20ins的两类药物EGFR-TKIs和mAbs具有不同的机制，二者联合使用时可实现胞内胞外双重EGFR阻断，较单类药物或可获得明显疗效增益，同时mAbs有可能弥补EGFR-TKIs治疗窗口较窄的短板^[86]^。近年EGFR-TKIs和mAbs联合靶向EGFR ex20ins的探索百花齐放，mAbs联合含铂化疗也获得了可喜的成绩。但与此同时，联合疗法也增加了3级及以上TRAE的发生，在临床应用中仍需权衡其疗效与潜在风险。

#### 3.3.1 阿法替尼联合西妥昔单抗

阿法替尼单药治疗EGFR ex20ins NSCLC患者的ORR低于10%^[36]^，在II期单臂临床试验^[87]^中与西妥昔单抗联合使用时可见明显疗效增益，18周ORR为32%，中位PFS为5.5个月，最常见TRAE为腹泻（70%）、皮疹（65%）和皮肤干燥（59%），54%的患者出现≥3级TRAE。

#### 3.3.2 奥希替尼联合耐昔妥珠单抗/JMT101

在奥希替尼联合疗法探索中，80 mg/d奥希替尼联合耐昔妥珠单抗^[88]^治疗化疗后进展EGFR ex20ins NSCLC患者，PFS达6.9个月，38%的患者出现≥3级TRAE，皮疹（21%）为主。

奥希替尼（160 mg, qd）联合JMT101的Ib期临床试验^[80]^中，经治EGFR ex20ins NSCLC患者ORR达36.4%，中位PFS达8.2个月，疗效受突变亚型影响较大，螺旋内插入突变明显优于近环突变和远环突变（ORR: 75% vs 36.7% vs 28.6%），可观察到较好颅内活性（ORR: 25%）。安全性上，最常见TRAE为皮疹（76.9%）、腹泻（63.6%），62%的患者出现≥3级TRAE。在纳入含铂类化疗失败患者的II期临床试验^[89]^中ORR可达50%，但高达73%的患者出现≥3级TRAE，主要为皮疹（32%）、腹泻（10%），导致了5%患者永久停药。2024年3月FDA批准用于EGFR ex20ins患者一线治疗^[90]^。

#### 3.3.3 埃万妥单抗联合化疗

III期PAPILLON研究^[91]^中，埃万妥单抗联合化疗较单一化疗显著提高了中位ORR和PFS（ORR：73% vs 47%；PFS：11.4 vs 6.7个月），但联合疗法造成3级及以上TRAE增加，主要包括中性粒细胞减少（33%）、贫血（11%）和皮疹（11%），7%的患者终止治疗。

### 3.4 其他药物

溴他替尼^[92]^、波奇替尼^[93]^和热休克蛋白90抑制剂Luminespib^[94]^疗效表现不佳，ORR仅为0%-17%。此外还有如BLU-451等正在进行I期临床试验的药物，将在表1中列出。

**表1:** EGFR ex20ins的相关靶向药物临床试验

Name of agents	Type of agents	Trial phase	Patient population	Treatment regimen	ORR (%)	Median PFS (mon)	Mainly grade≥3 TRAE (%)	Reference	Clinical identifier
Osimertinib	EGFR-TKI	Phase II	Without other EGFR-TKIs and bispecific antibodies pre-treated advanced EGFR ex20ins NSCLC	160 mg, qd	28	6.8	Diarrhea (4), rush (4), CPK increased (8)	^[43]^	NL6705
		Phase II	Pretreated advanced EGFR ex20ins NSCLC	160 mg, qd	25	9.7	Anemia (10), fatigue (10), prolonged QT interval (10), respiratory failure (5)	^[44]^	NCT03191149
Fumonertinib	EGFR-TKI	Phase Ib	Pre-treated advanced EGFR ex20ins NSCLC	240 mg, qd for the first line therapy; 160 mg/240 mg, qd for sequential therapy	69	10.7	Not disclosed	^[50]^	NCT04858958
		Phase III	Treatment-naive advanced EGFR ex20ins NSCLC	160 mg, qd; 240 mg, qd	Not disclosed	Not disclosed	Not disclosed	^[55]^	NCT05607550
Mobocertinib	EGFR-TKI	Phase I/II	Platinum-based chemotherapy pre-treated advanced EGFR ex20ins NSCLC	160 mg, qd	28	7.3	Diarrhea (21), nausea (4), stomatitis (4), lipase increased (4)	^[58]^	NCT02716116
		Phase III	Treatment-naive advanced EGFR ex20ins NSCLC	Mobocertinib 160 mg, qd vs Platinum-based chemotherapy	32 vs 30	9.59 vs 9.63	Diarrhea (20 vs 1), anemia (6 vs 10), increased lipase (6 vs 0), stomatitis (4 vs 0)	^[61]^	NCT04129502
Sunvozertinib	EGFR-TKI	Phase II	Platinum-based chemotherapy pretreated advanced EGFR ex20ins NSCLC	300 mg, qd	61	Not disclosed	Blood CPK increased (17), diarrhoea (8), anaemia (6)	^[66]^	NCT05712902
		Phase II	Treatment-naive advanced EGFR ex20ins NSCLC	200 mg, qd or 300 mg, qd	71.4	Not disclosed	Not disclosed	^[67]^	NCT03974022, NCT05559645
		Phase III	Treatment-naive advanced EGFR ex20ins NSCLC	Sunvozertinib vs Platinum-based doublet chemotherapy	Not disclosed	Not disclosed	Not disclosed	^[69]^	NCT05668988
Ziparlertinib	EGFR-TKI	Phase I/IIa	Pretreated advanced EGFR ex20ins NSCLC	5 dose levels, from 30 mg, bid to 150 mg, bid	38.4	10	Anemia (9.6), AST increased (4), diarrhea (3)	^[72]^	NCT04036682
YK-029A	EGFR-TKI	Phase I	Treatment-naive advanced EGFR ex20ins NSCLC	5 dose levels, from 50 mg, qd to 250 mg, qd	73.1	9.3	Diarrhea (6.5), hypokalemia (6.5), anemia (3.7)	^[74]^	NCT05767866
		Phase III	Treatment naive advanced EGFR ex20ins NSCLC	YK-029A vs Platinum-based chemotherapy	Not disclosed	Not disclosed	Not disclosed	^[69]^	NCT05767892
PLB-1004	EGFR-TKI	Phase I	Treatment-naive advanced EGFR ex20ins NSCLC	11 dose levels, from 10 mg, qd to 480 mg, qd	57.7	Not disclosed	Diarrhea (13.5), rash (6.6)	^[75]^	CTR20201030
		Phase II	Treatment-naive advanced EGFR ex20ins NSCLC	Not disclosed	Not disclosed	Not disclosed	Not disclosed	^[69]^	NCT06015503
		Phase III	Treatment-naive advanced EGFR ex20ins non-squamous NSCLC	PLB-1004 vs Platinum-based chemotherapy with or without Sintilimab	Not disclosed	Not disclosed	Not disclosed	^[69]^	NCT06281964
BEBT-109	EGFR-TKI	Phase I	Previously treated advanced EGFR ex20ins NSCLC	120 mg, qd; 180 mg, qd; 120 mg, bid	44.4	8	Diarrhea (22.2), rash (5.6)	^[77]^	CTR20192575
Amivantamab	Monoclonal aitibody	Phase I	Platinum pre-treated EGFR ex20ins NSCLC	1050 mg (weight<80 kg), 1400 mg (weight≥80 kg)	40	8.3	Hypokalemia (5), rash (4), pulmonary embolism (4), diarrhea (4), neutropenia (4)	^[81]^	NCT02609776
Afatinib plus Cetuximab	Combination therapy	Phase II	Advanced EGFR ex20ins NSCLC	Afatinib 40 mg, qd plus Cetuximab 500 mg/m^2^, q2w	32	5.5	Diarrhea (14), rash (14), dry skin (14)	^[87]^	NCT03727724
Osimertinib plus Necitumumab	Combination therapy	Phase I	Platinum-based chemotherapy pre-treated advanced EGFR ex20ins NSCLC	Osimertinib 80 mg, qd plus Necitumumab 800 mg d1+d8, q21d	22.2	6.9	Rash (21)	^[88]^	NCT02496663
Osimertinib plus JMT101	Combination therapy	Phase Ib	Pre-treated advanced EGFR ex20ins NSCLC	JMT101 6 mg/kg, q2w plus Osimertinib 160 mg, qd	36.4	8.2	Rash (21.3), diarrhea (10.7)	^[80]^	NCT04448379
		Phase II	Platinum-based chemotherapy pre-treated advanced EGFR ex20ins NSCLC	JMT101 6 mg/kg, q2w plus Osimertinib 160 mg, qd	50	Not disclosed	Rash (32), oral mucositis (11), diarrhea (10)	^[89]^	NCT05132777
Amivantamab plus chemotherapy	Combination therapy	Phase III	Treatment-naive advanced EGFR ex20ins NSCLC	Amivantamab plus Platinum-based chemotherapy	73	11.4	Neutropenia (33), leukopenia (11), rash (11)	^[91]^	NCT04538664
Tarloxotinib	EGFR-TKI	Phase II	Platinum-based chemotherapy pre-treated advanced EGFR ex20ins NSCLC	150 mg/m^2^, q1w	0	NA	Prolonged QTc (34.8), rash (4.3), diarrhea (4.3)	^[92]^	NCT03805841
Poziotinib	EGFR-TKI	Phase I/II	Platinum-based chemotherapy pre-treated advanced EGFR ex20ins NSCLC	16 mg, qd	14.8	4.2	Rash (28), diarrhea (26), stomatitis (9), paronychia (6)	^[93]^	NCT03318939
Luminespib	Hsp90 inhibitor	Phase II	Pretreated advanced EGFR ex20ins NSCLC	70 mg/m^2^, q1w	17	2.9	Ocular toxicity (3), hypertension (10), hypophosphatemia (7)	^[94]^	NCT01854034
BLU-451	EGFR-TKI	Phase I	Advanced EGFR ex20ins NSCLC	Not disclosed	Not disclosed	Not disclosed	Not disclosed	^[69]^	NCT05241873
BAY2927088	EGFR-TKI	Phase I	Pre-treated advanced EGFR ex20ins NSCLC	Not disclosed	Not disclosed	Not disclosed	Not disclosed	^[69]^	NCT05099172
FWD1509	EGFR-TKI	Phase I/II	Pre-treated advanced EGFR ex20ins NSCLC	Not disclosed	Not disclosed	Not disclosed	Not disclosed	^[69]^	NCT05068024
AP-L1898/JS111	EGFR-TKI	Phase I/II	Advanced EGFR ex20ins NSCLC	Not disclosed	Not disclosed	Not disclosed	Not disclosed	^[69]^	NCT04993391
HS-10376	EGFR-TKI	Phase I/II	Advanced EGFR ex20ins NSCLC	Not disclosed	Not disclosed	Not disclosed	Not disclosed	^[69]^	NCT05435274
Aumolertinib/Almonertinib	EGFR-TKI	Phase III	Treatment-naive EGFR ex20ins NSCLC	Aumolertinib vs Platinum-based chemotherapy	Not disclosed	Not disclosed	Not disclosed	^[69]^	NCT04951648

EGFR: epidermal growth factor receptor; TKI: tyrosine kinase inhibitot; CPK: creatione phosphokinse; AST: aspartate transaminase; ORR: objective response rate; PFS: progression-free survival; TRAE: treatment related adverse event; NSCLC: non-small cell lung cancer; NA: not applicable; Hsp90: heat shock protein 90.

## 4 总结与展望

随着计算机模型和NGS技术的发展，我们对EGFR ex20ins的理解加深，不同突变亚型的检出率得到了提高、多种靶向药物研究欣欣向荣，但仍旧面临不少问题亟待解决：（1）EGFR ex20ins的罕见性导致临床试验难以招募到足够的患者，且无法覆盖EGFR ex20ins全部的突变谱，有可能导致统计结果的偏倚；（2）当前药物研发数据虽好，但仍缺乏大型RWS数据进行验证；（3）假设本文所涉及的药物成功进入指南一线推荐疗法，其后续治疗方案的最佳选择、是否需要随一线治疗而灵活变化欠缺探讨；（4）PAPILLON等试验展示了联合疗法的潜力，但最佳治疗顺序和组合尚不明晰；（5）新兴药物如抗体药物偶联物，通过mAbs作为载体将小分子细胞毒性药物以靶向方式高效运输至肿瘤细胞，在EGFR突变患者上获得初步成效^[95]^，提供了治疗方案的新可能，但在EGFR ex20ins患者中的应用尚待研究；（6）不同突变亚型下游信号通路是否存在异质性导致EGFR ex20ins原发EGFR-TKIs耐药也仍不明确^[6]^；（7）研究^[10]^中发现EGFR ex20ins的不同突变亚型的强异质性导致对治疗的敏感程度不一，针对不同突变亚型对治疗方案更进一步分层细化仍存在巨大的进步空间。尽管如此，通过精准检测和个体化治疗，临床上有望为EGFR ex20ins患者提供更有效的治疗。
